# Anemia and risk for cognitive decline in chronic kidney disease

**DOI:** 10.1186/s12882-016-0226-6

**Published:** 2016-01-28

**Authors:** Manjula Kurella Tamura, Eric Vittinghoff, Jingrong Yang, Alan S. Go, Stephen L. Seliger, John W. Kusek, James Lash, Debbie L. Cohen, James Simon, Vecihi Batuman, Juan Ordonez, Gail Makos, Kristine Yaffe

**Affiliations:** VA Palo Alto Geriatric Research and Education Clinical Center, 3801 Miranda Ave., Palo Alto, CA 94304 USA; Division of Nephrology, Stanford University School of Medicine, Palo Alto, CA USA; Department of Epidemiology and Biostatistics, University of California San Francisco, San Francisco, CA USA; Division of Research, Kaiser Permanente Northern California, Oakland, CA USA; Department of Medicine, University of Maryland School of Medicine, Baltimore, MD USA; National Institute of Diabetes and Digestive and Kidney Diseases, Bethesda, MD USA; Department of Medicine, University of Illinois Chicago, Chicago, IL USA; Department of Medicine, University of Pennsylvania, Philadelphia, PA USA; Department of Nephrology and Hypertension, Glickman Urologic and Kidney Institute, Cleveland Clinic, Cleveland, OH USA; Department of Medicine, Tulane University Medical School, New Orleans, LA USA; Division of Nephrology, Kaiser Permanente Northern California, Oakland, CA USA; St. John Providence Medical Center, Detroit, MI USA; Departments of Psychiatry and Neurology, University of California San Francisco, San Francisco, CA USA; for the CRIC Study Investigators, Philadelphia, PA USA

**Keywords:** Cognitive decline, CKD, Anemia

## Abstract

**Background:**

Anemia is common among patients with chronic kidney disease (CKD) but its health consequences are poorly defined. The aim of this study was to determine the relationship between anemia and cognitive decline in older adults with CKD.

**Methods:**

We studied a subgroup of 762 adults age ≥55 years with CKD participating in the Chronic Renal Insufficiency Cohort (CRIC) study. Anemia was defined according to the World Health Organization criteria (hemoglobin <13 g/dL for men and <12 g/dL for women). Cognitive function was assessed annually with a battery of six tests. We used logistic regression to determine the association between anemia and baseline cognitive impairment on each test, defined as a cognitive score more than one standard deviation from the mean, and mixed effects models to determine the relation between anemia and change in cognitive function during follow-up after adjustment for demographic and clinical characteristics.

**Results:**

Of 762 participants with mean estimated glomerular filtration rate of 42.7 ± 16.4 ml/min/1.73 m^2^, 349 (46 %) had anemia. Anemia was not independently associated with baseline cognitive impairment on any test after adjustment for demographic and clinical characteristics. Over a median 2.9 (IQR 2.6–3.0) years of follow-up, there was no independent association between anemia and change in cognitive function on any of the six cognitive tests.

**Conclusions:**

Among older adults with CKD, anemia was not independently associated with baseline cognitive function or decline.

**Electronic supplementary material:**

The online version of this article (doi:10.1186/s12882-016-0226-6) contains supplementary material, which is available to authorized users.

## Background

Cognitive impairment affects up to one-third of patients with advanced chronic kidney disease (CKD) and end-stage renal disease (ESRD) [1, 2]. The prevalence of cognitive impairment increases early in the course of kidney disease, and several studies suggest CKD increases the risk for cognitive decline independent of age, diabetes and hypertension [3, 4]. However, the mechanisms leading to increased risk for cognitive decline among patients with CKD remain poorly understood.

In the general population, anemia is associated with increased risk for cognitive decline and dementia. In prospective studies, anemia has been associated with a 41–61 % increased risk for dementia [5–7]. Anemia is common among patients with CKD [8, 9], due in part to reduced erythropoietin production as kidney function declines. Some, but not all studies have identified a cross-sectional association between anemia and cognitive function in patients with ESRD. [2, 10] Previously, we found that lower hemoglobin (Hb) concentration was cross-sectionally associated with a higher prevalence of cognitive impairment using a single measure of cognitive function in the Chronic Renal Insufficiency Cohort (CRIC), a large, racially diverse cohort of adults with CKD [11]. Prospective studies of the association between anemia and change in cognitive function in patients with CKD are lacking.

We examined whether anemia was independently correlated with cross-sectional and longitudinal measures of cognitive function in adults with CKD. To address this question, we used data collected in the CRIC study, which included annual comprehensive cognitive assessments in a subset of older adults.

## Methods

### Study design and recruitment

The CRIC Study is a prospective observational cohort study designed to evaluate risk factors for progression of CKD among adults with moderate to advanced CKD. The study design, methods and baseline characteristics of study participants have been previously described [12, 13]. From June 2003 through May 2008, we recruited 3939 persons aged 21–74 years from seven clinical centers across the United States. Participants met age-based estimated glomerular filtration rate (eGFR) criteria: 20–70 ml/min/1.73 m^2^ for ages 21–44 years, 20–60 ml/min/1.73 m^2^ for ages 45–64 years, and 20–50 ml/min/1.73 m^2^ for ages 65–74 years. Exclusion criteria included diagnosis of polycystic kidney disease, pregnancy, recent immunosuppression for kidney disease, coexisting disease likely to affect survival, prior receipt of dialysis or organ transplant, or institutionalization (including residence in nursing homes).

### Ethics, consent and permissions

Institutional Review Boards at all clinical sites approved the study protocol and all participants provided written informed consent (Additional file 1).

### Assessment of cognitive function

Beginning in 2006, 825 participants aged 55 or older from four of the seven CRIC clinical centers were enrolled in an ancillary study (the CRIC COG Study). They underwent an annual battery of the six following cognitive function tests [14]. The Modified Mini-Mental State Exam (3MS) is a test of global cognitive function with components for concentration, orientation, language, praxis, and memory [15]. The Trailmaking Test (Trails) A measures attention, visuospatial scanning, and motor speed. The Trailmaking Test (Trails) B primarily assesses executive function [16]. Category Fluency evaluates verbal production, semantic memory, and language [17]. The Buschke Selective Reminding Test measures verbal memory with delayed components [18]. The Boston Naming assesses language function by asking participants to name objects presented in pictures [17].

### Assessment of anemia and other covariates

At the baseline study visit and each annual visit thereafter, a complete blood count was performed. For these analyses, we used the Hb measurement corresponding to the first cognitive battery assessment. Anemia was defined according to the World Health Organization criteria of Hb <13 g/dL in men or Hb <12 g/dL in women [19]. Red cell mean corpuscular volume was categorized as <80, 80–99, and ≥100 fL for descriptive purposes.

Socio-demographic and clinical characteristics were assessed at the baseline visit and updated annually. We defined diabetes by participant self-report, use of medications for diabetes, or fasting blood glucose of ≥126 mg/dL. We defined hypertension by participant self-report, use of medications for high blood pressure, or a seated blood pressure of ≥140/80 mm Hg. We defined coronary heart disease as participant self-report of a myocardial infarction, angina or coronary revascularization procedure. We defined cerebrovascular disease as participant self-report of a stroke. We defined peripheral vascular disease as participant self-report of claudication, amputation, or revascularization procedure of the extremities. We defined alcohol use as participant self-report of current or former use of beer, wine or liquor. Erythropoietin use in the previous 30 days was ascertained by medication review conducted by CRIC Study personnel. Serum creatinine concentration and cystatin C concentration were measured annually at the central study laboratory. We calculated the estimated glomerular filtration rate (eGFR) with an equation derived from the CRIC Study, using the annual serum creatinine and cystatin C measurements corresponding to the first cognitive function assessment [20].

### Statistical analyses

Continuous variables were expressed as means ± standard deviations (SDs) and compared using t-tests. Categorical variables were expressed as proportions and compared using the chi-squared test. We defined clinically significant cognitive impairment for each test separately, as a score one standard deviation (SD) or worse than the mean [21]. We estimated the association between anemia and baseline cognitive impairment using logistic models. The first model adjusted for demographic characteristics - age, sex, race, education and CRIC clinical center. The second model adjusted for demographic characteristics in addition to chronic health conditions -diabetes, hypertension, coronary artery disease, peripheral vascular disease, stroke, and alcohol use. The third model additionally adjusted for eGFR.

Next, we determined the association between anemia at baseline and annualized visit-to-visit changes in cognitive scores on each test, using mixed effects models with unstructured residual correlation matrix to account for within-subject correlation of the repeated changes. Cognitive assessments obtained after the development of ESRD were not included in longitudinal models. We constructed three models, as we did for the cross-sectional analysis, sequentially adjusting for baseline cognitive function and demographic characteristics, chronic health conditions, and eGFR. In sensitivity analyses, we excluded individuals receiving erythropoietin stimulating agents for treatment of anemia. We also conducted sensitivity analyses evaluating Hb as a continuous measure. All analyses were conducted using SAS 9.4 (Cary, NC).

## Results

**Fig. 1:**
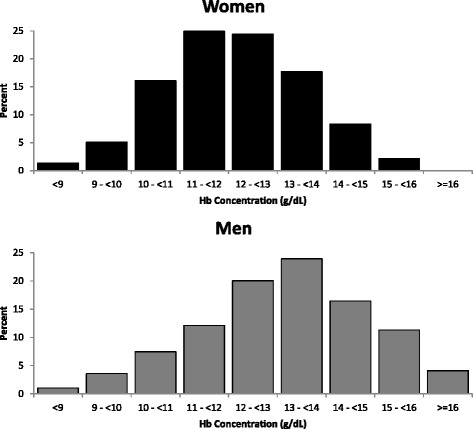
Distribution of hemoglobin concentration among 762 participants age ≥55 in the Chronic Renal Insufficiency Cohort

Of the 825 participants enrolled in the CRIC COG study, 26 were missing baseline Hb values and 37 were missing key covariates, resulting in an analytic cohort of 762 participants. The sample had a mean age of 64.4 ± 5.6 years and mean eGFR of 42.7 ± 16.4 ml/min/1.73 m^2^. Anemia was present in 349 (46 %) of participants. Compared to participants without anemia, participants with anemia were more likely to be black and have lower educational attainment (Table 1). In addition, participants with anemia were more likely to have diabetes, hypertension, coronary artery disease, peripheral vascular disease, and stroke, and less likely to currently use alcohol. Participants with anemia had lower eGFR and higher levels of albuminuria. The majority of participants with anemia had a mean corpuscular volume in the normocytic range (80–99 fL). There were 50 participants (6.6 %) receiving erythropoietin stimulating agents, including 38 (10.9 %) participants with anemia. The distribution of Hb values stratified by sex is shown in Fig. 1.

**Table 1:** Baseline characteristics of CRIC participants stratified by anemia status

Characteristics	No anemia	Anemia	*P*-value
	(*N* = 413)	(*N* = 349)	
Age, years			0.12
55–59	101 (24.5)	72 (20.6)	
60–69	231 (55.9)	188 (53.9)	
70–79	81 (19.6)	89 (25.5)	
Sex			0.37
Female	196 (47.5)	177 (50.7)	
Male	217 (52.5)	172 (49.3)	
Race			<0.001
White	262 (63.4)	125 (35.8)	
Black	120 (29.1)	206 (59.0)	
Other/Unknown	31 (7.5)	18 (5.2)	
Hispanic ethnicity			0.69
Hispanic	11 (2.7)	11 (3.2)	
Other/Unknown	402 (97.3)	338 (96.8)	
Education			<0.001
<High School	37 (9.0)	75 (21.5)	
High School Graduate	76 (18.4)	80 (22.9)	
Some College	300 (72.6)	194 (55.6)	
Diabetes mellitus	148 (35.8)	236 (67.6)	<0.001
Hypertension	353 (85.5)	337 (96.6)	<0.001
Prior coronary artery disease	108 (26.2)	110 (31.5)	0.10
Prior peripheral vascular disease	20 (4.8)	41 (11.7)	<0.001
Prior stroke	39 (9.4)	58 (16.6)	<0.01
Alcohol Use			<0.001
Current	293 (70.9)	165 (47.3)	
Former/Never	120 (29.1)	184 (52.7)	
Estimated GFR (ml/min/1.73 m^2^)	48.6 (15.6)	35.7 (14.5)	<0.001
Albuminuria (mg/g)			<0.001
<30	235 (56.9)	119 (34.1)	
≥30	168 (40.7)	106 (62.5)	
Unknown	10 (2.4)	12 (3.4)	
Mean corpuscular volume (fL)			<0.001
<80	16 (3.9)	32 (9.2)	
80–99	392 (94.9)	301 (86.2)	
>=100	5 (1.2)	15 (4.3)	
Unknown	0 (0.0)	1 (0.3)	
Erythropoietin use	12 (2.9)	38 (10.9)	<0.001

In analyses adjusted for demographic characteristics, anemia was associated with higher odds of cognitive impairment on the Trails A at baseline, but not significantly associated with impairment on other cognitive tests (Table 2). After adjustment for age, sex, race, education, CRIC clinical center, diabetes, hypertension, coronary artery disease, peripheral vascular disease, stroke, alcohol use, and eGFR, there was no significant association between anemia and cognitive impairment on any of the six cognitive measures.

**Table 2:** Association of anemia with baseline cognitive impairment among participants age ≥55 in the Chronic Renal Insufficiency Cohort

Cognitive test	N	Odds ratio (95 % CI)		
		Model 1	Model 2	Model 3
Modified Mini-Mental State Exam	762	1.48 (0.85–2.59)	1.45 (0.81–2.61)	1.59 (0.86–2.94)
Category Fluency	760	1.49 (0.92–2.41)	1.50 (0.91–2.48)	1.43 (0.85–2.40)
Buschke Delayed Recall	753	1.19 (0.77–1.86)	1.19 (0.75–1.89)	1.06 (0.66–1.71)
Boston Naming	760	1.34 (0.83–2.16)	1.21 (0.73–2.00)	1.25 (0.73–2.14)
Trailmaking Test A	759	1.93 (1.12–3.32)	1.72 (0.98–3.04)	1.72 (0.95–3.10)
Trailmaking Test B	759	1.42 (0.89–2.28)	1.19 (0.73–1.96)	1.16 (0.69–1.94)

Model 1 is adjusted for age, sex, race, education and CRIC clinical center

Model 2 is adjusted for age, sex, race, education, diabetes, hypertension, coronary artery disease, peripheral vascular disease, stroke, alcohol use, and CRIC clinical center

Model 3 is adjusted for age, sex, race, education, CRIC clinical center, diabetes, hypertension, coronary artery disease, peripheral vascular disease, stroke, alcohol use, and estimated glomerular filtration rate

There were 70 participants who did not complete any follow-up cognitive assessments. The remaining 692 participants were followed for a median of 2.9 years (IQR 2.6–3.0). There was no significant association between anemia and change in cognitive function on the 3MS, Buschke Delayed Recall, Boston Naming and Trails A tests in the partially adjusted or fully adjusted models (Table 3). After adjustment for baseline cognitive function, age, sex, race, education and CRIC clinical center, there was a significant association between anemia and more pronounced decline on the Trails B test, and between anemia and less pronounced decline on the Category fluency test (Table 3). After additional adjustment for chronic health conditions the associations between anemia and change in the Trails B and Category Fluency tests were attenuated and no longer significant. Further adjustment for eGFR did not change these findings.

**Table 3:** Adjusted mean annual change in cognitive scores between anemic vs non-anemic participants. A positive parameter estimate denotes a larger decline in cognitive function for the Modified Mini-Mental State Exam, Category Fluency, Buschke Delayed Recall and Boston Naming tests. For the Trailmaking Test A and B, a negative parameter estimate denotes a larger decline in cognitive function

Cognitive test	N	Mean annual change (SE)	Parameter estimate for anemia vs. non-anemia (SE)	*P*-value
Modified mini-mental state exam	692			
Model		0.21 (0.05)	0.190 (0.12)	0.11
Model 2			0.170 (0.13)	0.17
Model 3			0.130 (0.13)	0.31
Category fluency	690			
Model 1		0.20 (0.06)	−0.280 (0.11)	0.01
Model 2			−0.200 (0.11)	0.08
Model 3			−0.140 (0.12)	0.22
Buschke delayed recall	692			
Model 1		0.19 (0.03)	−0.030 (0.06)	0.58
Model 2			−0.040 (0.06)	0.56
Model 3			−0.060 (0.06)	0.37
Boston naming	691			
Model 1		0.07 (0.01)	0.040 (0.02)	0.08
Model 2			0.040 (0.02)	0.08
Model 3			0.040 (0.02)	0.06
Trailmaking test A	685			
Model 1		−0.93 (0.27)	0.070 (0.46)	0.88
Model 2			−0.210 (0.48)	0.66
Model 3			−0.200 (0.48)	0.68
Trailmaking test B	685			
Model 1		−2.36 (0.61)	−3.220 (1.52)	0.03
Model 2			−2.330 (1.62)	0.15
Model 3			−1.830 (1.67)	0.27

*SE* standard error

Model 1 is adjusted for age, sex, race, education and CRIC clinical center

Model 2 is adjusted for age, sex, race, education, diabetes, hypertension, coronary artery disease, peripheral vascular disease, stroke, alcohol use, and CRIC clinical center

Model 3 is adjusted for age, sex, race, education, CRIC clinical center, diabetes, hypertension, coronary artery disease, peripheral vascular disease, stroke, alcohol use, and estimated glomerular filtration rate

In sensitivity analyses excluding individuals receiving erythropoietin stimulating agents, the results were similar. For example, the fully adjusted association between anemia and change in the Category Fluency test was −0.10 ± 0.27 (*p*-value = 0.22), and between anemia and change in the Trails B test was −0.71 ± 3.98 (*p*-value 0.27). Similarly, we found no significant association between Hb, evaluated as a continuous measure, with changes in cognitive function in fully adjusted models.

## Discussion

In a well-characterized cohort of older adults with CKD studied prospectively, we did not find an independent association between anemia and baseline cognitive function or the rate of cognitive decline over nearly 3 years. Our findings were consistent across multiple domains of cognitive function including global cognition, executive function, language and memory. These results do not support the hypothesis that anemia is an independent risk factor for cognitive decline in older persons with CKD.

There is limited information regarding the relationship between anemia and cognitive function in patients with CKD. In the full CRIC cohort of more than 3500 participants, we previously reported a cross-sectional association between Hb, evaluated as a continuous measure, and impairment on the 3MS, a measure of global cognition. Most previous studies of CKD and anemia were conducted in patients with ESRD receiving maintenance dialysis, and many of these studies were conducted shortly after the introduction of erythropoietin for treatment of anemia. Early studies posited that treatment of anemia with erythropoietin might have salutary effects on brain function among patients with ESRD receiving dialysis. Most of these studies evaluated brain evoked potentials as an end-point; however a few also assessed cognitive function. For example, in an uncontrolled study of 24 patients, treatment with erythropoietin over 12 months increased hematocrit from 23.7 to 36.0 %, and was associated with an improvement in attention and executive function [22]. The absence of a control group in these studies limits the ability to distinguish learning effects from a causal effect of anemia treatment. Contemporary studies conducted in hemodialysis patients with less severe anemia, most of whom were receiving treatment with erythropoietin, have not found a strong relation between anemia and cognitive function. In one study of 338 patients with ESRD assessed with a cognitive battery, Hb <11 g/dl was associated with cognitive impairment in unadjusted analyses, but not in adjusted analyses [2]. In a cross-sectional study of 109 patients with ESRD, unadjusted Hb concentration was correlated with global cognition, but not with executive function [10]. In another cross-sectional study of 383 patients with ESRD, there was no association between Hb concentration and global cognition or executive function [23].

Compared to prior studies, the current study utilized a longitudinal study design. Although anemia was common in this cohort, treatment with erythropoietin was infrequent, reflective of current practice guidelines [24]. There are several possible explanations why anemia might be associated with dementia and cognitive decline in the general population [5–7], but not among patients with CKD. Compared to studies in the general population, the prevalence of anemia in the CRIC Study cohort was higher, but the follow-up period was shorter, and this may have been insufficient to identify clinically significant changes in cognitive function. We censored patients at ESRD to eliminate the potential confounding effects introduced by the changes in health status and treatment of anemia surrounding this critical transition period. It is possible that the risk for cognitive decline is increased only for patients with severe anemia, that is, a Hb concentration well below the WHO definition. This seems unlikely, however, since prior studies in the general population also utilized the WHO definition of anemia.

Another possible explanation of our findings is that the association between anemia and cognitive impairment is confounded by nutritional deficiencies, such as B12 deficiency, and that these nutritional deficiencies are a more common cause of anemia in the general population. We lacked measures of B12, folate, and iron stores in the current study. Nevertheless, the majority of participants in the CRIC COG Study had a normocytic anemia, which would not be typical for isolated B12 or iron deficiency anemia. Erythropoietin deficiency has also been suggested as a mechanistic link between anemia and cognitive decline, based on animal models demonstrating erythropoietin receptors in the brain and uncontrolled clinical studies of patients treated with erythropoietin stimulating agents. Given that erythropoietin deficiency plays a major role in the anemia of CKD, this pathway seems unlikely to be involved based on the results of the current study. Finally, it may be that anemia is not causally related to cognitive decline in patients with CKD, but that it is a marker for other conditions which affect cognition. This conclusion is suggested by the observation that demographic and clinical characteristics largely attenuated the association between anemia and baseline cognitive function and cognitive decline.

Our study has several strengths, including the multi-center, prospective design, diverse study population, use of a cognitive battery to assess cognitive function, and ability to account for a number of important confounding factors. We acknowledge several limitations. First, the follow-up period was short. Second, our participants (volunteers for a long-term research study) may be at lower risk for cognitive decline than the broader population with CKD because they are selected for the ability to comply with study visits and they may be more motivated to pursue healthy behaviors. Both of these factors may have limited the power to detect changes in cognitive function and biased our study towards the null. Thus, a more subtle relationship between anemia and cognitive function may have gone undetected because of a type II error. We also lacked information about nutritional factors associated with anemia such as B12, folate, and iron.

## Conclusions

Although anemia was common among older adults with CKD, it was not independently associated with baseline cognitive function or the rate of cognitive decline. For now, the mechanisms contributing to cognitive decline in patients with CKD remain elusive.

## Additional file

Additional file 1:
**Supplemental File.** (DOCX 12 kb)

## Abbreviations

CKD: chronic kidney disease
CRIC: chronic renal insufficiency cohort
eGFR: estimated glomerular filtration rate
ESRD: end stage renal disease
Hb: hemoglobin
