# Supplementary material for: Anemia and risk for cognitive decline in chronic kidney disease
Source: BMC Nephrol. 2016 Jan 28;17:13. doi: 10.1186/s12882-016-0226-6 (PMC4730636; doi:10.1186/s12882-016-0226-6)
Supplement: Additional file 1: — Supplemental File. (DOCX 12 kb) [file 12882_2016_226_MOESM1_ESM.docx]

Supplementary File

IRB approvals: University of Pennsylvania (707819 and 807882), University Hospitals of Cleveland (02-03-04), MetroHealth Medical Center (IRB03-00052), Cleveland Clinic Foundation (5969), University of Illinois (2003-0149), Kaiser Permanente/UCSF (CN-01AGo-02-H).
